# Circulating Extracellular Vesicles: The Missing Link between Physical Exercise and Depression Management?

**DOI:** 10.3390/ijms22020542

**Published:** 2021-01-07

**Authors:** Edna Soares, Julie Reis, Mariana Rodrigues, Carlos Fontes Ribeiro, Frederico C. Pereira

**Affiliations:** 1 Institute of Pharmacology and Experimental Therapeutics/IBILI, Faculty of Medicine, University of Coimbra, 3000-548 Coimbra, Portugal; juliehelenereis@gmail.com (J.R.); mfgmr12@gmail.com (M.R.); fontes.ribeiro@gmail.com (C.F.R.); 2 Faculty of Medicine, Coimbra Institute for Clinical and Biomedical Research (iCBR), University of Coimbra, 3000-548 Coimbra, Portugal; 3 Center for Innovative Biomedicine and Biotechnology (CIBB), University of Coimbra, 3004-504 Coimbra, Portugal; 4 Clinical Academic Center of Coimbra (CACC), 3004-504 Coimbra, Portugal

**Keywords:** depression, physical exercise, extracellular vesicles, circulating biomarkers

## Abstract

Depression is associated with an increased risk of aging-related diseases. It is also seemingly a common psychological reaction to pandemic outbreaks with forced quarantines and lockdowns. Thus, depression represents, now more than ever, a major global health burden with therapeutic management challenges. Clinical data highlights that physical exercise is gaining momentum as a non-pharmacological intervention in depressive disorders. Although it may contribute to the reduction of systemic inflammation associated with depression, the mechanisms underlying the beneficial physical exercise effects in emotional behavior remain to be elucidated. Current investigations indicate that a rapid release of extracellular vesicles into the circulation might be the signaling mediators of systemic adaptations to physical exercise. These biological entities are now well-established intercellular communicators, playing a major role in relevant physiological and pathophysiological functions, including brain cell–cell communication. We also reviewed emerging evidence correlating depression with modified circulating extracellular vesicle surfaces and cargo signatures (e.g., microRNAs and proteins), envisioned as potential biomarkers for diagnosis, efficient disease stratification and appropriate therapeutic management. Accordingly, the clinical data summarized in the present review prompted us to hypothesize that physical exercise-related circulating extracellular vesicles contribute to its antidepressant effects, particularly through the modulation of inflammation. This review sheds light on the triad “physical exercise–extracellular vesicles–depression” and suggests new avenues in this novel emerging field.

## 1. Introduction

Depression is a mood disorder affecting about 300 million people worldwide [1]. This picture may even worsen in the upcoming years due to the psychosocial effects of the COVID-19 pandemic, producing stress, anxiety and depression in the general population [2,3,4]. Depressive disorders are characterized by clear-cut changes in mood, interests and pleasure, cognitive deficits and suicide as the worst case scenario [5]. Currently used diagnostic and therapeutic approaches are quite disappointing. Diagnostic methodologies rely on a cluster of highly variable symptoms using (semi)structured interviews, such as the Diagnostic and Statistical Manual of Mental Disorders (DSM) [6,7], and the most commonly used therapy relies on antidepressant drugs, often associated with a lack of patient adherence, poor treatment response and several pharmacological side effects [8]. Consequently, physical exercise (PE) has been explored as a non-pharmacologic therapeutic option, strongly validated by clinical trials, reviews, meta-analysis and appropriate guidelines [9,10,11,12]. Regular PE brings numerous physiological and psychological benefits, along with the lower propensity to develop chronic age-related disorders (e.g., cardiovascular, metabolic, neurodegenerative and psychiatric) [13,14]. Still, 27.5% of the worldwide population was inactive in 2016 [15], and efforts should be made to reverse that. Consistently, the World Health Organization (WHO) recommends at least 150 or 75 min of moderate or vigorous intensity PE per week [16], and the American Psychiatric Association and the National Institute for Health and Care Excellence also provided evidence-based guidelines on PE use as non-pharmacological therapy for psychiatric disorders, mostly to improve depression symptoms [11,17].

**Figure 1:**
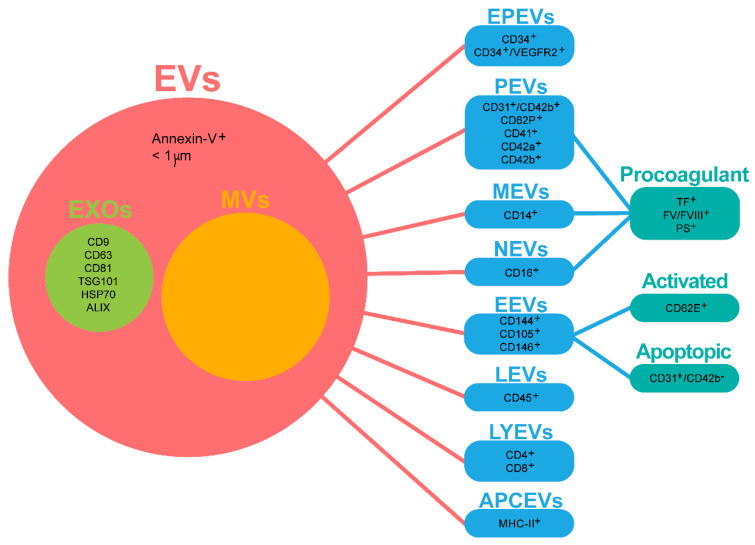
Nomenclature and cell-specific subtypes of circulating extracellular vesicles. In this review, focus will be given to extracellular vesicles (EVs), which include both exosomes (EXOs) and microvesicles (MVs). EXOs were distinguished when appropriate markers were identified. This decision was supported by the known overlap in size/marker definitions between EXOs and MVs (also called ectosomes or microparticles) and by the fact that most studies here included referred to MVs as broad EVs with size < 1 μm and/or annexin-V^+^. Additionally, cell-specific subtypes of the most common circulating EVs were categorized and uniformed according to the studies here reviewed. For a full list of other markers, please see [51,52,62]. APCEVs, antigen-presenting cell-derived vesicles, EEVs, endothelial-derived vesicles, EPEVs, endothelial progenitor cell-derived vesicles, EVs, extracellular vesicles, EXOs, exosomes, FV/FVII, coagulation factors V/VII, LEVs, leucocyte-derived vesicles, LYEVs, lymphocyte-derived vesicles, MEVs, monocyte-derived microvesicles, NEVs, neutrophil-derived microvesicles, PEVs, platelet-derived vesicles, PS, phosphatidylserine and TF, tissue factor.

PE is usually categorized as aerobic or anaerobic according to the intensity, interval, types of recruited muscle fibers and the energy metabolism. Both types are known to exert cardiovascular benefits, but it is still not clear if one is better than the other [18]. Nevertheless, it is widely accepted that aerobic PE has several positive effects on neurodegenerative disorders and mental health [10,12,19,20,21,22]. Although some possible underlying mechanisms have already been associated with increased circulating serotonin [23] and neurotrophic factors [24], reduced systemic inflammation [25] and brain mitochondrial bioenergetics modulation [26], mechanisms of PE action in the brain remain poorly understood. Excitingly, the PE-induced release of myokines (coined “exerkines”, all the myokines—muscle-secreted factors—released in response to endurance exercise) by the skeletal muscle may play a major role in muscle–organ tissue crosstalk, possibly mediating the systemic adaptions to PE, including the brain [27]. More interestingly, PE has been recently described to trigger a rapid release of extracellular vesicles (EVs) from a variety of cells into the circulation [28,29]. These vesicles may enclose those “exerkines” or any other active mediators of intercellular communication, such as surface receptors and genetic material [30]. EVs may differ in size, biogenesis/release pathways, composition or function. Therefore, they are categorized in three main groups: (1) exosomes (EXOs), (2) microvesicles (MVs) and (3) apoptotic bodies. EXOs are nano-sized vesicles (30–150 nm) released upon multivesicular body (MVB; also called late endosomes) fusion with the plasma membrane [31]. Accordingly, EXOs are enriched in endosomal markers (e.g., ALIX and TSG101), targeting/adhesion proteins (e.g., integrins and intercellular adhesion molecules), tetraspanins (e.g., CD63 and CD81), heat shock proteins (HSPs) (e.g., HSP70) and specific lipid molecules (e.g., ceramide and phosphatidylserine (PS)) [32]. MVs are larger EVs (0.1–1 µm) that result from phospholipidic reorganization of the cell membrane and cytoskeletal protein contraction, leading to outward blebbing and fission of the plasma membrane [33,34]. MVs are essentially enriched with the cytoskeleton, trafficking and adhesion proteins (e.g., matrix metalloproteinases and annexins), HSPs and glycoproteins (e.g., GPIb and GPIIb-IIIa) [29,32]. Lastly, apoptotic bodies are the largest EVs (500–4000 nm) released from the plasma membrane during programmed cell death [33,35]. In this review, focus will be given to EVs (<1 μm, annexin-V^+^), which include both EXOs and MVs. EXOs were distinguished based on appropriate markers (Figure 1).

EVs have been identified in several biological fluids, including blood plasma [36] and serum [37], urine [38], saliva [39], synovial fluid [40], cerebrospinal fluid (CSF) [41], amniotic fluid [42] and breast milk [43] and, therefore, are regarded as potential biomarkers. Blood is an excitingly non-invasive alternative to capture EVs from the brain, which may be a promising platform for “liquid biopsies” in neurodegenerative or psychiatric disorders, namely depression. In fact, the lack of an accurate diagnosis represents a major obstacle to effective depression management [44,45,46]. Circulating biomarkers may reflect the pathological changes in the central nervous system (CNS), enabling more accurate depression diagnosis and stratification [47,48] and unveiling exciting perspectives for the monitoring of the effectiveness of PE in depression [27,46,49]. Indeed, blood EV concentrations and cargo signatures (proteins, RNAs, microRNAs (miRNAs), non-coding RNAs and mRNAs) are coming to light as valuable biomarkers [50]. EVs derived from different cells may have different contents and, consequently, play different functions [51]. Although most cells are able to secrete EVs into the bloodstream, the most common sources are circulatory system cells [29,52], such as endothelial cells (EEVs), platelets (PEVs), lymphocytes (LYEVs) and monocytes (MEVs) [53,54,55] (Figure 1). Accordingly, EVs must be isolated and further characterized. Ultracentrifugation is the golden standard for EV isolation amongst other commonly used methodologies, such as size exclusion chromatography (SEC), filtration, solubility/precipitation and affinity interaction-based methods [56]. Flow cytometry (FC), transmission electron microscopy (TEM), nanoparticle tracking analysis (NTA) and immune affinity assays (Western blot (WB) and enzyme-linked immunosorbent assay (ELISA)) are the most frequently used characterization techniques [57]. Expectedly, several challenges come with biofluids, including viscosity, density, the presence of EV-sized proteins/lipoproteins and EVs from different cell sources. Therefore, pure EV subtype isolation remains an ambitious task [58]. The International Society for Extracellular Vesicles (ISEV) proposed Minimal Information for Studies of Extracellular Vesicles (“MISEV”), including both general (three positive and one negative marker) and single vesicle characterization (two different and complementary techniques) guidelines [59]. Additionally, high-throughput techniques (e.g., mass spectrometry and next-generation sequencing) and data analysis in light of integrated databases (e.g., EVpedia and ExoCarta) are emerging as mandatory for the proclamation of EVs as validated biomarkers [60,61].

Overall, the present review hypothesizes that PE-associated circulating EVs contribute to the PE-induced amelioration of depressive symptoms. Moreover, these circulating EVs can be a diagnostic and prognostic candidate tool for depression. We encourage and suggest new avenues in this novel emerging field, as we believe that clarification of the underlying mechanisms of beneficial effects of PE may represent a step further in the implementation of PE as a non-pharmacological strategy in depression.

## 2. Search and Selection Criteria to Avoid Confounding Factors

**Figure 2:**
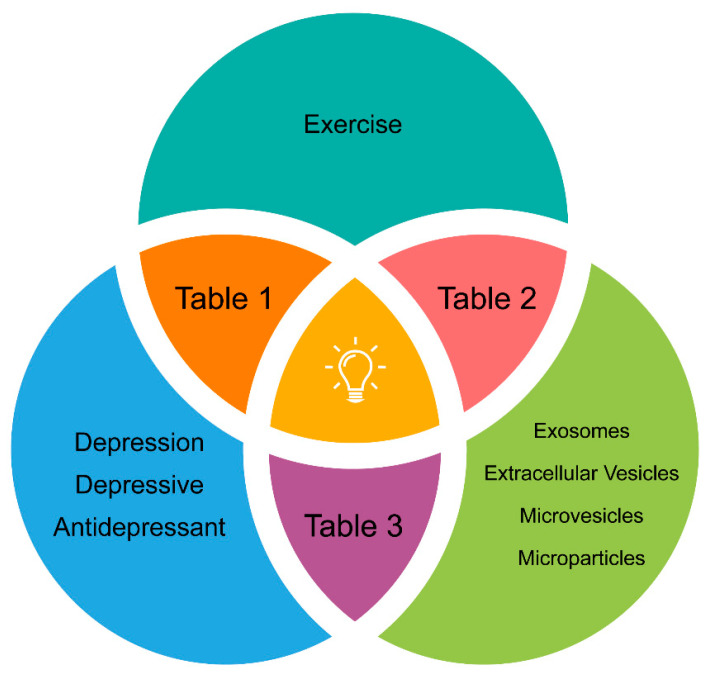
Keywords from the PubMed online database search. Results are shown for each combination of keyword search including clinical trials published over the last 5 years (2014–2019) according to the inclusion/exclusion criteria described in the manuscript. The results are summarized in Table 1, Table 2 and Table 3, as illustrated.

Figure 2 illustrates the search strategy in the PubMed database, which was focused on clinical studies published in the last five years. A combination of the following keywords in the titles/abstracts was employed: (“exercise”) AND (“exosomes” OR “extracellular vesicles” OR “microvesicles” OR “microparticles”) AND (“depression” OR “depressive” OR “antidepressant”). Studies featuring PE + depression, PE + EVs and EVs + depression are summarized in Table 1, Table 2 and Table 3, respectively. Several inclusion/exclusion criteria were applied to bring uniformity and to avoid possible confounding factors on the outcome results. Pilot trials with *n* ≤ 10 were excluded. Only supervised and monitored moderate-to-vigorous aerobic exercise was included in Table 1 and Table 2. As we were interested in the role of PE as therapy *per se*, studies where PE was tested as an add-on therapy to antidepressant medication were excluded from Table 1. Therefore, only trials where less than one-third of the study population was taking medication for depression were included, attenuating the contribution of antidepressant medication to the antidepressant effect seen in studies using physical exercise. To measure the real impact of PE on circulating EVs (not biased by any medical condition), only reports on healthy subjects were included in Table 2. Table 1 and Table 3 include reports focused on subjects with a primary diagnose of a depressive disorder (DSM-5 or similar), with no other known non-psychiatric comorbidities. Lastly, Table 2 and Table 3 include studies focused on circulating EVs from the peripheral blood. As circulating EVs comprise a heterogeneous collection of EVs mostly released from several circulating cell types, Figure 1 elucidates the nomenclature used in the present review.

## 3. Evidence Supporting the Triad Physical Exercise-Extracellular Vesicles-Depression

Brain functions may be influenced by several internal and external factors, including PE, aging and stress [63,64,65,66,67]. Indeed, aerobic PE is now recognized as an effective treatment for depressive disorders [10,12,19]. Unlike antidepressant medication, it is an inexpensive, non-invasive and mostly free of adverse side effect means to prevent and correct several mechanisms associated with depression-related mortality [12]. Accordingly, the COVID-19 pandemic-induced negative changes in physical activity were associated with higher depression, anxiety and stress symptoms [2]. PE may affect the brain at the structural, functional and molecular levels [68], with the recent literature suggesting that the release of proinflammatory cytokines, neurotrophic factors and miRNAs are able to regulate neurogenesis, dendritic and synaptic remodeling, subsequently affecting the hippocampal neuroplasticity and depressive phenotypes [63,64,68,69,70,71,72,73]. Additionally, PE may increase myokines, which can exert autocrine or paracrine/endocrine biological effects, controlling processes such as metabolism, angiogenesis and inflammation [62]. Nonetheless, robust evidence of these PE-related mechanisms is still lacking. This may explain why PE is still underutilized as a therapeutic intervention in depression. Meanwhile, recent and growing research proposes that EVs released in the course of PE may be active players in the beneficial systemic adaptions to PE, including in the brain [27,51,52,62,74]. In 2018, Safdar and Tarnopolsky [27] proposed that both myokine and “exerkine” cargoes within EXOs are instrumental in promoting interorgan crosstalk, thus modulating the systemic adaptations to PE. Evidence for such an argument was readily supported by other authors over the last couple of years [49,51,52,62,74]. Excitingly, the emerging potential of circulating EVs in the pathogenesis, diagnosis and treatment of psychiatric disorders, including depression, sparked our interest [44,45,46]. EVs are known to cross the blood–brain barrier (BBB) and, thus, may provide a relatively non-invasive way to obtain information from the brain (peptides or miRNA biomarkers) and may function as delivery vehicles to target brain dysfunction [44,46]. Although there is some evidence regarding circulating proteins [75] or miRNAs [44] as putative biomarkers for depression, scientific studies on the effects of PE-associated circulating EVs on either depression onset, progression or amelioration are still missing. Nonetheless, it is tempting to hypothesize that PE-associated circulating EVs may contribute to the biological effects of PE on depression and depressive symptoms. Therefore, subsequent sections aim to provide a detailed review of the current knowledge that supports our hypothesis.

### 3.1. Aerobic Exercise and Depression

In this section, the effect of aerobic PE in depressive disorder symptoms will be discussed (Table 1). Importantly, during the PE/depression keywords search (Figure 2), we found that most of the studies involved participants with other known comorbidities (e.g., metabolic and cardiovascular diseases, cancer and fibromyalgia), and only the ones without any non-psychiatric comorbidities were included in Table 1. As expected, all confirmed the positive benefits of regular aerobic PE practice on depression [9,76,77,78,79]. Remarkably, the effects were evident for different PE settings and studied populations, namely regarding age and sex. Specifically, PE interventions ranged from short-term (three weeks) to long-term (12 weeks) protocols and included low- (LI), medium- (MI) and high (HI)-intensity running, cycling, aerobics class or circuit training. Although only studies testing moderate-to-vigorous PE intensities were included, some trials reported the inclusion of lower-intensity PE groups, and those results may also be considered for discussion. Participants ranged from mostly female to only male and from adolescents to older adults. Remarkably, other PE-induced improvements, besides depressive symptomatology, were also reported in trials described in Table 1. Namely, improved cardiopulmonary fitness [9], an increased work capacity over time [77], increased health-related quality of life and physical activity rates [79], slight decrease in panic and fear, increased seeking of “basic emotion command systems” in the brain [76] and improved neural indices of conflict monitoring and cognitive control processes [78]. Finally, the ”Regassa Project” is the most robust study published over the last five years on PE/depression [77] and, thus, deserves further consideration. In this trial, 946 patients diagnosed with mild-to-moderate depression were randomly assigned to compare the effectiveness of PE, internet-based cognitive-behavioral therapy (ICBT) and treatment as usual (TAU) over 12 weeks. Moreover, the PE arm comprised three different forms and intensities of physical activity implemented at the same frequency (three times a week): LI comprised a one-hour yoga class, MI comprised a one-hour aerobics class and HI comprised a one-hour aerobics/strength/balance class. Only the MI and HI PE interventions included aerobic PE. Nevertheless, both the PE arm as a whole and the ICBT group were more effective than TAU, showing larger improvements in depressive symptoms. TAU consisted of the standard treatment for depression prescribed by primary care physicians (ICBT, supportive counseling or antidepressant medication or a combination thereof). The authors concluded that PE and ICBT represent promising non-stigmatizing therapeutic alternatives for mild-to-moderate depression. Given the strength of the study, four subgroup reanalyzes on its data were also found in the PubMed search [80,81,82,83]. For instance, Helgadottir et al. [80] segregated PE groups according to exercise intensity and performed a comparative analysis including a TAU group (TAU, LI PE, MI PE and HI PE). Interestingly, they found that both LI yoga classes or HI aerobics were more effective than TAU or MI PE at the 12-month follow-up. The differences among the PE groups were fairly small, and these findings provided some indications that physicians may appropriately prescribe exercise for depressive disorders at any intensity level, possibly taking individual preferences into account.

Inflammation seems to be the centerpiece of the intricate network of mechanisms possibly mediating the beneficial effects of PE in depression [64,65,66,72,84]. PE can promote molecular changes that switch a chronic proinflammatory state, as evidenced by the increased circulating proinflammatory cytokines observed in some depressive patients [64,66], into an anti-inflammatory state, both systemically and in the brain [64,65]. Several mechanisms have been postulated to mediate such an anti-inflammatory response. Savitz suggested that the kynurenine pathway (KP) may establish the connection between PE, inflammation and psychiatric disorders, due to the KP broad impact on emotion, cognition, pain, metabolic function and aging [66]. He also discussed the clinical utility of kynurenines as biomarkers for mood disorders, as KP dysregulation may weaken the neuroplasticity and, therefore, the impact on psychiatric illnesses. Two secondary analyses of the “Regassa Project” focusing on the PE arm as whole studied inflammatory mediators. One study found a positive correlation between the decreased interleukin-6 (IL-6) plasma levels and the decreased depression severity induced by PE [81]. The other one failed to see changes in the kynurenine and kynurenic acid (kynA) plasma levels [82]. However, the KP still remains a putative mechanism that needs to be further explored in this context. The hypothalamic–pituitary–adrenal (HPA) axis is another key component of the physiological network triggered by a prolonged, stress-induced proinflammatory state [45,64,72]. Sometimes, such chronic stress may culminate in glucocorticoid resistance (a non-responsive immune system), leading to the coexistence of HPA axis hyperactivity and increased inflammation, preponderant factors for depression severity or recurrence [64]. The elevation of glucocorticoids also reduces neurotrophins, thus hampering neuroplasticity [72]. The preclinical data suggest that PE can potentially rescue stress-impaired neuroplasticity, modulating the adaptive response of the HPA axis to stress and by regulating the neurotrophic factors, including brain-derived neurotrophic factor (BDNF) [72]. Indeed, BDNF is a marker of neuroplasticity: for example, BDNF is required for the proper development and survival of dopaminergic, GABAergic, cholinergic and serotoninergic neurons [67,68,72,85]. Additionally, the last reanalysis of the “Regassa Study” (Table 1) aimed to explore whether the BDNF variation valine (Val)-to-methionine (Met) substitution at codon 66 (Val66Met) and childhood adversity (a broad term referring to circumstances that pose a serious threat to a child’s physical or psychological well-being, specifically including parents’ divorce or death, financial problems or the existence of friction in the family) predicted the treatment response [83]. Depression risk/recovery was reported to depend on a functional single nucleotide polymorphism (SNP) in BDNF (rs6265; G to A polymorphism encoding a valine (Val)-to-methionine (Met) substitution at codon 66 (Val66Met)) [86,87]. This SNP impairs intracellular BDNF trafficking and further secretion [88] and was also suggested to mediate depressive symptom changes in response to PE [89]. These authors also looked at childhood adversity, because it has been reported to increase depression susceptibility by interacting with BDNF genetic variations. However, this secondary analysis found that BDNF Val66Met or childhood alone did not predict a treatment response [88]. In fact, only Met allele carriers that were not exposed to childhood adversity but were currently on antidepressants showed a higher treatment response (to PE) than Val homozygotes. Met carriers also had higher serum mature BDNF, suggesting that they would benefit more from PE treatment but only if they were not exposed to early child adversity. De Assis et al. [85] even argued that BDNF synthesis and reuptake dynamics resemble the functioning of metabolic systems of aerobic energy generation. Additionally, increased BDNF concentrations are correlated to the amount of aerobic energy required during PE in a dose-dependent manner. These authors further suggested that the peroxisome proliferator-activated receptor gamma coactivator 1-alpha (PGC1-α) may be a link between aerobic metabolism and neuroplasticity. Other authors similarly proposed that PE improves the PGC1-α/BDNF pathway (muscle/brain) through the signaling of circulating irisin, an exercise-induced hormone. This “exerkine” may strengthen synapses and reduce oxidative stress and inflammatory cytokine synthesis and release. On the other hand, PGC1-α is also involved in the balance of the KP (reduced quinolinic acid), leading to reduced glutamatergic neurotoxicity and, consequently, reduced neuroinflammation [64,66]. These mechanisms may further contribute to the neuroprotective and antidepressant effects of PE [84]. Overall, studies investigating the influence of PE on depression should address the interplay of all these possible mediators.

**Table 1:** Aerobic physical exercise effects on depressive symptoms of subjects with depressive disorder (Diagnostic and Statistical Manual of Mental Disorders (DSM-5) or similar).

Study Year	Sample	Physical Exercise	Severity of Depression	Major Findings	
Chronic Aerobic Physical Exercise					
2019[9]	Sedentary subjects with MDD (22 ± 2 years; *n* = 11)	Frequency and duration: 3 days/week; 12 weeks (chronic)Intensity: MI incrementalType: cycling, running, elliptical use, rowing or a combinationTreatment groups: 1 (PE)	HAMD_17_	↓ depression scores after PE; time spent in target HRR zone correlated with ↓ anxiety symptoms	
2017[78]	Sedentary university students with non-psychotic MDD (mostly female; 21 ± 2 years; *n* = 15)	Frequency and duration: 3 days/week; 8 weeks (chronic)Intensity: LI and MI Type: running or cyclingTreatment groups: 2 (LI PE; MI PE)	BDI-II score	↓ depressive symptoms after MI PE (not LI PE)	
2016[76]	Males with depression (24.5 years; *n* = 9–11)	Frequency and duration: 3 days/week; 3 weeks (chronic)Intensity: LI, MI and HIType: running or cyclingTreatment groups: 3 (LI PE; MI PE; HI PE)	HAMD and MADRS	↓ depressive symptoms after both MI and HI PE (not LI PE)	
2015[79]	Adolescents with depression (CDI-2 score > 14, 15 ± 1 years; *n* = 43–44)	Frequency and duration: 2 days/week; 6 weeks (chronic)Intensity: MI intervalType: circuit training (abdominal, back, ball arm-based exercises, bouncing, static and dynamic balance, squat and cycling)Treatment groups: 2 (PE + TAU; TAU)	CDI-2 score	↓ depressive symptoms at 6-months follow-up; unchanged depressive symptoms immediately after treatment	
2015[77]	Sedentary subjects with mild to moderate depression (PHQ-9 score > 9, mostly female; 43 ± 12 years; *n* = 310)	Frequency and duration: 3 days/week; 12 weeks (chronic)Intensity: LI, MI and HIType: yoga class (LI), aerobics (MI and HI)Treatment groups: 3 (PE (LI, MI and HI); ICBT and TAU)	PHQ-9 score and MADRS	↓ depressive symptoms for PE and ICBT (compared to TAU)	
				↓ depression severity for LI PE (compared to TAU and MI PE) and for HI PE (compared to MI PE)4 subgroups (TAU (*n* = 310), LI PE (*n* = 106), MI PE (*n* = 105) and HI PE (*n* = 99)) [80]	Secondary Analyses (2017)
				↓ IL-6 was paralleled by ↓ depression severity after PE1 subgroup (PE (*n* = 116)) [81]	
				↑ treatment response to PE for Met allele carriers without exposure to childhood adversity but currently on antidepressants (compared to Val homozygotes); ↑ serum BDNF for Met carriers; BDNF Val66Met or childhood adversity did not predict treatment response5 subgroups (2—BDNF variation Val66Met (Val/Val, *n* = 377 and Met carriers, *n* = 170) and 3—childhood adversity (no, mild-to-moderate and high)) [83]	
				Unchanged kynurenine and kynurenic acid plasma levels1 subgroup (PE (*n* = 117)) [82]	

**Notes:** TAU commonly represents psychosocial and/or antidepressant treatment; physical exercise protocols are described excluding the warm-up; secondary analyses are included in “Major Findings” of the corresponding original studies. BDNF, brain-derived neurotrophic factor, BDI-II, Beck depression inventory-second edition, CDI-2, children’s depression inventory—2nd Version, HAMD_17_, 17-item Hamilton rating scale for depression, HI, high-intensity, HRR, heart rate reserve, ICBT, internet-based cognitive–behavioral therapy, LI, low-intensity, MADRS, Montgomery–Asberg depression rating scale, MDD, major depressive disorder, MI, moderate-intensity, PE, physical exercise, PHQ-9, 9-item patient health questionnaire, TAU, treatment as usual, IL-6, interleukin-6, Met, methionine, Val, valine, Val66Met, Val-to-Met substitution at codon 66,↑, increase and ↓, decrease.

### 3.2. Aerobic Exercise and Extracellular Vesicles

The data summarized in Table 2 represent the most recent trials assessing circulating EV profiles after moderate-to-vigorous PE, focused only on healthy subjects, to avoid the impact of comorbidities on the EV dynamics triggered by PE. In fact, the circulating EV signature following PE in clinical populations, mostly with cardiovascular risk, was already presented in recent comprehensive reviews [51,52,90]. Wilhelm et al. [51] found similarities between blood EVs and the cytokine adjustments to PE. While acute PE may lead to a transient increase of cytokines and EVs derived from platelets in circulation, long-term PE practice may decrease endothelial EV linked to vascular damage at rest in analogy to inflammatory cytokines. Overall, the studies reviewed herein focus on circulating EV responses to acute and chronic PE.

Most trials tested the acute effects of a single cycling or running bout in circulating EVs (tested time points: immediately and 30 or 60 min post-PE) [54,55,91,92]. Others were interested in EV release dynamics and collected blood at several time points, from the baseline (pre) to two days after PE intervention [49,52,53,93,94,95]. Studies collecting blood during PE practice reported seemingly contradictory findings. While Shill et al. [95] found no alterations during MI continuous or HI interval PE, Brahmer et al. [53] reported a significant gradual increase in EVs from pre- over an interim time point to post-PE during a cycling bout to exhaustion. Three major reasons may explain these apparent divergent findings. First, different physical activities were compared: running versus cycling. Second, EVs were sorted using different markers: CD34^+^ and CD62^+^ versus CD9^+^, CD63^+^ and CD81^+^. Third, the EVs were collected at different time points: while the first study collected blood halfway through the PE [95], the second study collected blood samples specifically at a respiratory exchange ratio of 0.9 (RQ 0.9) [53]. This aerobic parameter corresponds to a submaximal exercise level in typical medium-intensity aerobic activity for both trained and untrained persons [96]. However, since the fold changes in EV signal intensity were marginal in the Brahmer study, one should not overstate the apparent differences between these studies. A pilot study testing incremental cycling or running until exhaustion found different EV kinetics according to the PE type [97]. While EVs significantly increased immediately after cycling, declining within 90 min post-PE, the circulating EV response to treadmill running was more moderate, but the EV increase was more sustained over time. Additionally, they found that EV release initiated in the early phase during the incremental cycling exercise (aerobic) before the individual anaerobic threshold (rise of lactate). This suggests that the aerobic exercise triggers a rapid release of EVs into the circulation, which may have proangiogenic potential, as demonstrated in vitro [98]. In fact, some studies herein reviewed reported increased circulating EVs immediately after PE (0–2 min; Table 2) [49,53,54,55,94,99]. The majority of these authors provided a detailed analysis and reported that EVs originated from several cellular origins increased after PE (Figure 1): EVs originated from endothelial cells (EEVs; total (CD144^+^, CD105^+^ or CD146^+^), activated (CD62E^+^) and apoptotic (CD31^+^/CD42b^-^)) either globally [94] or sex-specific [55]; endothelial progenitor cells (EPEVs, sex-specific) [55]; platelets (PEVs); lymphocytes (LYEVs) and antigen-presenting cells (APCEVs) [53]. On the other hand, no changes in EEVs in the total population (total, activated and apoptotic) [55]; PEVs and MEVs [99]; neutrophil-derived vesicles (NEVs) [99] or EPEVs [95] were reported or even a reduction in activated EEVs [95], MEVs or NEVs [94] acutely following PE. This heterogeneity of results may reflect a diversity of experimental conditions, including the following: male versus female, sedentary versus active subjects and different type and intensity of exercise (running versus marathon versus cycling or MI versus HI or continuous versus interval versus incremental to exhaustion—please see cautionary notes on modulating factors section). During early recovery from PE, the results showed that EEVs (total, activated and apoptotic) [92] and procoagulant NEVs [99] were decreased when measured from 30 up to 180 min after PE. Additionally, immediately increased EV levels returned to their basal levels at later time points [49,94]. All these studies seem to have a major impact in the cardiovascular field. In fact, elevated EEVs, PEVs and EVs released by inflammatory cells are recognized as candidate biomarkers of vascular stress or cardiovascular diseases [52,90]. Specifically, elevated EEVs are thought to reflect vascular injury and, therefore, are considered a marker of endothelial dysfunction (early atherosclerosis and hypertension) [100], and increased LEVs, NEVs and PEVs are implicated in thrombotic events. Additionally, apoptotic EEVs reflect endothelial cell apoptosis as a consequence of endothelial-dependent vasodilation in patients with coronary artery disease [51,52,90].

Although the majority of the studies presented dealt with acute exercise, three studies focused on chronic PE. Two of these studies provided new insights into how chronic exercise affects the vascular thrombotic risk under hypoxic conditions [54,101]. These authors showed that an acute hypoxic exercise test (HET, 12% O_2_, 30 min) induced enhanced dynamic thrombin generation and increased circulating procoagulant NEVs [101] or MEVs together with elevated plasma concentrations of norepinephrine/epinephrine, myeloperoxidase and IL-6 [54] (Table 2). It is known that the release of procoagulant NEVs accelerates the pathogenesis of inflammatory thrombosis and atherosclerotic lesions that accumulate procoagulant MEVs, increasing the risk of atherothrombosis [102]. Both trials demonstrated that previous regular PE over five weeks was able to provide resistance to thrombotic risk factors provoked by hypoxia [54,101]. Remarkably, cycling training at either normoxic (NPE) or hypoxic (HPE) conditions suppressed the HET-induced release of procoagulant EVs and desensitized the extent of dynamic thrombin generation [101]. Nevertheless, regular HPE was the most effective intervention in increasing both the aerobic capacity and the resistance to the risk of vascular thrombosis induced by hypoxia when compared to NPE [54]. Consistently, reduced levels of circulating activated EEVs were found following regular PE over six months in another trial [103]. The circulating EEVs may be uptaken by tissues engaged in crosstalk communication triggered by PE [62]. Overall, some studies in Table 2 show that PE changes the circulating EVs profile, which confirms vascular, immune and inflammatory adaptations to PE [53,55,94,99].

**Table 2:** Acute and chronic aerobic physical exercise effects on circulating extracellular vesicles.

Study Year	Sample	Physical Exercise	Extracellular Vesicles	Major Findings
**Acute Aerobic Physical Exercise**				
2019 [92]	Fit females (pre-, peri- and post-menopausal; 45 ± 1 years; *n* = 11–13)	Frequency and duration: acute Intensity: MI Type: running	Blood collection: pre- and 30 min after PE Isolation and detection: cell-free plasma; FC (calibration beads) Characterization: FC	↓ EEVs (↓ activated and ↓ apoptotic) after PE; ↑ percentage of activated EEVs (but not apoptotic) after PE; no effect of menopausal status on EEVs
2019 [99]	Male subjects (23 ± 3 years; *n* = 15)	Frequency and duration: acute (participants completed one exercise and one control trial separated by at least 5 d in a randomized crossover design (www.randomization.com). Intensity: HI Type: running	Blood collection: pre-, 0 and 90 min after PE Isolation and detection: cell-free plasma; ultracentrifugation, FC Characterization: FC	Unchanged EV concentration or diameter (NTA); ↑ EV counts immediately after PE (FC); ↓ procoagulant PEVs/NEVs at 0/90 min after PE
2019 [53]	Male athletes (28 ± 4 years; *n* = 21)	Frequency and duration: acute Intensity: incremental to exhaustion Type: cycling	Blood collection: pre-, during and 2 min after PE Isolation and detection: cell-free plasma; size-exclusion chromatography, EXOs immuno-bead isolation (CD9^+^/CD63^+^/CD81^+^) Characterization: NTA, FC, proteomics	↑ EVs during PE (highest levels at peak PE); ↑ EXO markers, ↑ EEVs, ↑ PEVs, ↑ MEVs, LYEVs and ↑ APCEVs after PE; markers of muscle (SGCA) not detected
2018 [49]	Male subjects (27 ± 1 years; *n* = 11)	Frequency and duration: acute Intensity: incremental to exhaustion Type: cycling	Blood collection: pre-, 0 min and 4 h after PE Isolation and detection: cell-free plasma; ultracentrifugation Characterization: NTA, proteomics	↑ EVs after PE, returning to basal in 4 h; ↑ 322 proteins after PE (biogenesis and function of EXOs and small EVs and several biological processes, most notably the glycolytic pathway)
2018 [95]	Males and females (24 ± 5 years; *n* = 20)	Frequency and duration: acute Intensity: MI continuous or HI interval Type: running	Blood collection: pre-, during and after (0, 30, 60, 90 and 120 min) PE Isolation and detection: cell-free plasma; FC (calibration beads) Characterization: FC	Analysis of EVs at all seven times revealed no significant interaction; ↓ activated EEVs immediately after MI continuous PE (not HI interval); sex-differentiated analysis showed ↓ activated EEVs after MI PE only in women; unchanged EPEVs; menstrual cycle phase did not affect EVs
2018 [94]	Marathon runners (49 ± 6 years; *n* = 99)	Frequency and duration: acute Intensity: marathon running Type: running	Blood collection: pre-, 0 min and 2 d after PE Isolation and detection: cell-free plasma; FC Characterization: FC	↑ EEVs (↑ activated and ↑ apoptotic) and ↑ PEVs after marathon, returning to basal within 2 days; ↓ MEVs and ↓ LEVs after marathon, remaining ↓; number of participants’ marathon runs in total (but not running time) correlated with ↑ EEVs (activated and apoptotic)
2018 [93]	Male subjects (25 ± 4 years; *n* = 10)	Frequency and duration: acute Intensity: HI Type: cycling	Blood collection: pre, 5 min and 4 h after PE Isolation and detection: cell-free plasma; size exclusion columns Characterization: WB, qRT-PCR	EXO markers detected (CD63, HSP70); ↑ 12/29 target miRNA after PE (miR-1-3p, -16-5p and 222-3p coincident with muscle and plasma; miR-23a-3p, 208a-3p and -150-5p coincident with muscle; miR- 486-5p, 378a-5p, 126-3p coincident with plasma; and miR- 23b-3p, 451a and 186-5p only in EXOs), associated with myogenic differentiation, fibre identification, blood vessel formation and insulin responsiveness
2016 [55]	Active subjects (25 ± 1 years; *n* = 18)	Frequency and duration: acute Intensity: HI incremental Type: cycling	Blood collection: pre- and 0 min after PE Isolation and detection: cell-free plasma; FC (<1 μm) Characterization: FC	↑ activated EEVs in male and ↑ EPEVs in female after PE (unchanged for men and women combined); ↓ EPEVs baseline in female
2015 [91]	Sedentary and fit males (26 ± 5 years; *n* = 18)	Frequency and duration: acute Intensity: HI Type: running	Collection: pre- and 60 min after PE Isolation and detection: cell-free plasma; ultracentrifugation; density gradient separation Characterization: EXOs isolation kit, FC, WB, qRT-PCR	5% of EVs were SGCA^+^ (<1 µm); 60–65% of SGCA^+^ EVs were CD81^+^; ↑ miR-206/miR16 in SGCA^+^ EVs; ↑ miR-206 in TSG101/SGCA^+^ EVs; ↑ miR-181a-5p after PE; correlation between VO_2_ max and EVs miR-1, miR-133b, miR-206, miR-499 and miR-181a
**Chronic Aerobic Physical Exercise**				
2017 [103]	Sedentary African Americans (53 ± 1 years; *n* = 10–23)	Frequency and duration: 3 days/week; 6 months (chronic) Intensity: MI incremental Type: walking/jogging, stair stepping, cycling, rowing, arm and elliptical cross-training	Collection: pre- and after PE Protocol Isolation and detection: cell-free plasma; filtration, FC (<1 μm) Characterization: FC	↓ activated EEVs and unchanged total EEVs after PE
2015 [101]	Sedentary males (23 ± 1 years; *n* = 20)	Frequency and duration: 5 days/week, 5 weeks (chronic) Intensity: MI and HI interval Type: cycling	Blood collection: pre- and 0 min after HET (100 W under 12% O_2_ for 30 min); HET was performed 2 d before and 2 d after PE Isolation and detection: cell-free plasma; filtration/FC (annexin-V^+^, <1 µm) Characterization: FC	MI and LI PE attenuated HET-induced ↑ NEVs
2015 [54]	Sedentary males (21 ± 0.4 years; *n* = 40)	Frequency and duration: 5 days/week, 5 weeks (chronic) Intensity: MI Type: NE (21% O^2^) or HE (15% O^2^) cycling	Blood collection: pre- and 0 min after HET (100 W under 12% O2 for 30 min); HET was performed 2 d before and 2 d after PE Isolation and detection: cell-free plasma; FC (FSC, calibration beads, annexin-V^+^) Characterization: FC	↑ MEVs and ↑ procoagulant MEVs after acute HET, supressed (↓) after both NE and HE 5-wk PE protocol (more pronounced for HE)

**Notes:** “Microparticles” and “microvesicles” terminology was used by most authors to define EVs < 1 μm. Therefore, we used the generic nomenclature extracellular vesicles (EVs). We specifically used the terminology exosomes (EXOs) when EVs were characterized based on the appropriate markers. The markers and nomenclature used for different circulatory cell-derived EVs are illustrated in Figure 1; some studies did not mention participants’ physical conditions (sedentary, active or fit); if no other parameter is specified, decreased (↓) or increased (↑) “EVs” refer to vesicle concentration. FC, flow cytometry, FSC, forward scatter light, HE, hypoxic exercise, HET, hypoxic exercise test, HI, high-intensity; HSP70, heat-shock protein 70, IL-6, interleukin-6, LI, low-intensity, MI, moderate-intensity, NE, normoxic exercise, NTA, nanoparticle tracking analysis, PE, physical exercise, qRT-PCR, quantitative reverse transcription polymerase chain reaction, SGCA, α-sarcoglycan, TSG101, tumor susceptibility gene 101 protein, VO_2_ max, maximum rate of oxygen consumption, EEVs, endothelial EVs, PEVs, platelet EVs, LYEVs, lymphocyte EVs, APCEVs, antigen-presenting cell EVs and WB, Western blot.

Thereafter, several trials performed a detailed characterization of PE-associated circulating EVs regarding their surface and cargo properties (“exerkines”) (Table 2) [49,53,91,93]. These studies may further enhance the global characterization of PE-induced EV patterns after a single bout of running [91] or cycling [49,53,93]. Some authors asked if active muscle could release EVs carrying miRNAs potentially involved in muscle repair, regeneration and remodeling [91]. Their study was prompted by Baggish et al. [104] and Bye et al. [105] seminal findings that reported correlations between aerobic PE and blood miRNA modulation (miR-146a, -21, -210 and -222). Guescini et al. [91] found a positive correlation between aerobic fitness and miRNAs specifically expressed in muscle (“myomiRs”). Interestingly, the exosomal marker TSG101 and the muscle tissue marker α-sarcoglycan (SGCA) were enriched (WB) in the same isolation fraction, suggesting the presence of plasma EXOs originating from muscles. Additionally, 60–65% of those SGCA^+^ were also CD81^+^ (an EXO surface marker). Finally, circulating myomiRs were, at least in part, included in muscle-derived EVs (miR-206 “myomiR”, TSG101 and SGCA were enriched in the same fraction). Moreover, they increased the miR-206/miR-16 ratio (miR-16 is stably expressed in blood) in SGCA^+^ EVs compared to SGCA- EVs. Overall, these indicate that muscles also release EVs. However, a FC analysis revealed that only 1–5% of total circulating EVs were SGCA^+^ [91]. On the other hand, Brahmer et al. [53] found no muscle tissue marker after a single bout of cycling to exhaustion. Lastly, the VO_2_ max was also positively correlated with miR-1, -133b, -206, -499 (“myomiRs”) and -181a present in EVs. Therefore, these miRNAs were suggested as aerobic fitness biomarkers [91]. Another study also focused on the expression of 29 miRNAs (regulated by PE) in muscles, plasma and plasma EXOs following acute cycling [93]. These authors found that 12 miRNAs were increased in plasma EXOs (miR-1, -16 and -222 coincide with muscles and plasma; miR-23a, -208a and -150 coincide with muscles; miR-486, -378a and -126 coincide with plasma and miR-23b, -451a and -186 only in EXOs). Additionally, three miRNAs (miR-23a-3p, -208a-3p and -150-5p) were responsive to PE in muscles and EXOs but not plasma, which emphasizes the unique EXO miRNA signature (compared to free-circulating miRNA). All these miRNAs were mostly associated with myogenic differentiation, fiber identification, blood vessel formation and insulin responsiveness, in agreement with the known muscle, metabolic and cardiovascular adaptations to PE. A systematic proteomic characterization of EV signatures after PE was also conducted [49]. One hour of cycling led to the increase of 322 proteins mostly correlated with EXO biogenesis/function and the glycolytic pathway. Although the metabolic adaptations to PE remain to be studied, the vesicle trafficking of metabolic mediators may be the process by which tissues can share resources during high-energy demands (like aerobic PE). In particular, Whitham et al. [49] demonstrated that PE-induced circulating EVs had a propensity to localize in the liver of recipient mice and, also, transfer their cargo to target hepatocytes (in vitro). This is strongly suggestive that EVs mediate tissue crosstalk, possibly including other organs, such as the brain. Altogether, the impact of PE on the number, origin and cargo of circulating EVs suggests that these small vesicles are implicated in several biologic processes during exercise, including vascular, immune, muscle and metabolic mechanisms. Moreover, all these findings hint at the complex contribution of EVs to the claimed tissue crosstalk induced by exercise, given their systemic nature.

### 3.3. Depression and Extracellular Vesicles

Circulating EVs are gaining attention in psychiatric disorders diagnosis and therapy—namely, in depression [44,45,46]. Specifically, EXO involvement in brain synaptic plasticity, neuronal stress response, cell-to-cell communication and neurogenesis highlight their functional significance and emerging role in mental disorders [106]. Their ability to cross the BBB renders them useful as an accessible and relatively non-invasive way to obtain information from the CNS. EXOs are thought to cross the BBB through transcytosis, which could pose a limitation regarding the distinction of EXOs originating from the CNS or shed from the BBB. Additionally, it has been suggested that BBB and CNS endothelial cell markers also exist in circulating EXOs [107,108,109]. Those EXOs may reflect the pathophysiologic changes in the CNS, which could help to overcome the major burdens of psychiatric disorders: subjective diagnosis and prognosis [44,45,46]. Interestingly, neuronal-enriched systemic EVs (phosphorylated tau, amyloid β42 (Aβ42) and phosphorylated insulin receptor substrate 1 (IRS-1)) were recently reported as validated biomarkers for Alzheimer’s disease (AD) [110]. Coherently, candidate CSF protein biomarkers for MDD [75] and potential diagnostic miRNAs for depression [44,106] have already been suggested as well. Still, studies correlating these biomarkers with EVs in the context of depressive disorders remain limited and need to be expanded by the scientific community. The studies described in Table 3 were focused on young and adult subjects diagnosed with MDD [111,112] or in a current MDD episode [113,114]. Participants were either drug-free [111,114] or taking antidepressant/antipsychotic medication [112,113]. In line with what was already validated for AD, Nasca et al. reported an increased number of EXOs enriched for L1 cell adhesion molecules (L1CAM, predominantly expressed in the brain) in subjects with MDD, which, in turn, were enriched for the insulin receptor substrate-1 (IRS-1) [113]. Interestingly, these findings were further associated with subjects’ suicidality and anhedonia behaviors. On the other hand, a relationship between the expression of IRS-1 in L1CAM^+^ EXOs and systemic insulin resistance was only found in healthy controls (HCs), and sex differences were reported in the serine-312 phosphorylation of IRS-1 in L1CAM^+^ EXOs in the MDD group. These findings provide a starting point to determine whether this circulating EV fingerprint could reflect brain insulin resistance and might represent trait markers of MDD. Another study revealed that the top differentially expressed EXO miRNA (genome-wide analysis), upregulated hsa-miR-139-5p, had good performance in differentiating between MDD and HC subjects [111]. EXO hsa-miR-139-5p was also upregulated in an animal model of depression, resulting in the impairment of adult hippocampal neurogenesis and depressive-like behaviors, rescued by the intranasal injection of miR-139-5p antagomir. Additionally, the authors demonstrated that EXOs from MDD patients cause depressive-like behaviors in mice, reinforcing that EXO miRNAs have an active role in the pathogenesis of neuropsychiatric diseases. Recently, other authors suggested that the microbiome composition may also be a potential tool to distinguish psychiatric disorders, namely subjects with MDD and bipolar disorder (BD) [112]. Bacterial DNA of the *Prevotella* 2 and *Ruminococcaceae* UCG-002 genera found in serum EXOs were significantly more prevalent in MDD than either in BD or HC and, therefore, proposed as candidates for distinguishing these two psychiatric diseases. These microbial genera are known to influence intestinal mucosal immunity and barrier function, mediating the regulation of interleukin circulation and, consequently, systemic and neuroinflammation through the modulation of the microglia function [115]. *Prevotella* abundance has been observed in various inflammatory disorders, associated with T-helper type 17 immunity [116], and some evidence suggests a *Ruminococci* association with inflammatory bowel disease [117]. A functional analysis of the pathways revealed that the mineral absorption, Wnt signaling, Notch signaling, and chronic myeloid leukemia pathways were enhanced in the MDD group compared to the HCs. The apoptosis function differed between all three groups. An important finding was that the ascorbate and aldarate metabolism functions were significantly decreased in the MDD group. One should keep in mind that the antioxidant and anti-inflammatory properties of ascorbate may account for its putative therapeutic effects [118]. Microbiota–gut–brain communication is gaining attention in a myriad of diseases [115], and it is well-known that neuroinflammation and immune dysregulation are crucial factors in the pathophysiology of MDD [64,119,120]. Accordingly, Kuwano et al. [114] analyzed neuron-derived exosomes (NDE) in the peripheral blood of MDD patients and found that synaptophysin (SYP, a synaptic protein), tumor necrosis factor receptor 1 (TNFR1) and IL-34 (associated with MDD neuroinflammation) were strongly positively correlated with CD81 (EXO surface marker). Remarkably, this study is a pioneer in the suggestion that not only SYP and TNFR1 but, also, IL-34 could be important blood biomarkers for patients with MDD. Importantly, the TNFR1/CD81 ratio was positively correlated with depression severity and symptomatology. Consistently, similar findings are also emerging in preclinical studies. For example, Gómez-Molina et al. [121] reported that rat serum small EVs contained astrocyte-derived biomarkers of repetitive stress (e.g., aldolase C and glial fibrillary acidic protein (GFAP)).

All these findings represent a small body of evidence suggesting that the circulating EV signature may bring new insights to the context of depressive disorders and may bring psychiatric research into a bright new paradigm. The correlation of EVs with depression symptoms deserves further studies and represents a potential tool for personalized medicine strategies. Additionally, there is an ongoing clinical trial using EXOs to treat depression, anxiety and dementia (https://clinicaltrials.gov/ct2/show/NCT04202770). This further gives credence to the hypothesis whereby EVs could be a therapeutic strategy in depression.

**Table 3:** Depressive disorder (DSM-5 or similar) effects on circulation extracellular vesicles.

Study Year	Sample	Severity of Depression	Extracellular Vesicles	Major Findings
2020 [111]	Subjects with MDD (drug-free; 28 ± 1.7 years; *n* = 33) or Schizophrenia (drug-free; 29 ± 1; *n* = 36) and HC (29 ± 1; *n* = 46)	HAMD and MADRS	Blood collection: overnight fasting Isolation and detection: serum; SEC + centrifugation/concentration Characterization: qRT-PCR, miRNA-seq, bioinformatics	38 blood exosomal miRNAs differently expressed in MDD and HC, 24 were upregulated and 14 downregulated (from 351 miRNAs for differential analysis); axon guidance and development, dendrite, Wnt signaling pathway, neuron-to-neuron synapse and PI3K-Akt signaling pathway were closely related to MDD; ↑ hsa-miR-139-5p top differentially expressed between MDD and HC (specific for MDD, not Schizophrenia); hsa-miR-139-5p and a cluster of 15 miRNAs or 10 miRNAs with excellent performance to differentiate between MDD and schizophrenia (MDD-specific)
2020 [113]	Subjects with MDD in a current major depressive episode (43 ± 2 years; *n* = 64) and age and sex-matched HC (38 ± 2 years; *n* = 29)	HDRS-21	Blood collection: 6 h fasting, 6 h no PE Isolation and detection: plasma; precipitation + ultracentrifugation; WB expression array (CD63, CD81, ALIX, FLOT1, ICAM1, EpCam, ANXA5, and TSG101) Characterization: ExoCET (AChE activity), FC, ELISA	Unchanged EXOs between MDD and HC; ↑ L1CAM^+^ EXOs in MDD; ↑ IRS-1 in L1CAM^+^ EXOs in MDD (no sex or psychotropic medication differences); no between-group difference in pSer-IRS-1 in L1CAM^+^ EXOs (only sex differences in MDD group); IRS-1 in L1CAM^+^ EXOs correlated with systemic IR for HC but not MDD; no relationship between IRS-1 in L1CAM^+^ EXOs and depression severity in MDD; the highest IRS-1 in L1CAM^+^ EXOs correlated with depressed mood, feelings of guilt, suicidality and anhedonia (MDD)
2020 [112]	Subjects with MDD (34 ± 11; *n* = 30) or BD subjects (46 ± 10; *n* = 42) and HC (43 ± 6; *n* = 36)	HAMD and YMRS	Blood collection: no specifications Isolation and detection: serum; centrifugation + filtration (0.22 μm) Characterization: PCR, bioinformatics	EVs *Prevotella* 2 and *Ruminococcaceae* UCG-002 genera significantly more prevalent in MDD than BD or HC; apoptosis function differed between groups
2018 [114]	MDD Subjects in acute episode of MDD (drug-free, 31 ± 7; *n* = 34) and sex and age-matched HC (*n* = 34)	BDI-II	Blood collection: no specifications Isolation and detection: plasma; sandwich ELISA detection (CD81) Characterization: sandwich ELISA	Neuron-related blood biomarkers moderately to strongly positively correlated with CD81; ↑ IL-34/CD81 in MDD (compared to control); SYP/CD81 and TNFR1/CD81 positively correlated with depression scores

**Notes:** “Microparticles” and “microvesicles” terminology was used by most authors to define EVs < 1 μm. Therefore, we used the generic nomenclature extracellular vesicles (EVs). We specifically used the terminology exosomes (EXOs) when EVs were characterized based on appropriate markers. The markers and nomenclature used for different circulatory cell-derived EVs are illustrated in Figure 1. AChE, acetylcholinesterase, BD, bipolar disorder, BDI-II, Beck depression inventory-second edition, ELISA, enzyme-linked immunosorbent assay, FC, flow cytometry, HAMD, Hamilton rating scale for depression, HC, healthy controls, HDRS-21, Hamilton Depression Rating Scale, IL-34, interleukin-34, IRS-1, insulin receptor substract-1, L1CAM, L1 cell adhesion molecule, MADRS, Montgomery–Asberg depression rating scale, MDD, major depressive disorder, PCR, polymerase chain reaction, qRT-PCR, quantitative reverse transcription polymerase chain reaction, SEC, size exclusion chromatography, SYP, synaptophysin, TNFR1, tumor necrosis factor receptor 1, WB, Western blot, YMRS, 11-item Young Mania Rating Scale and ↑, increase.

## 4. Cautionary Note on Modulating Factors

Several internal and external factors may modulate the response of both depression or circulating EVs to PE. EVs are released upon cell stress, and their circulating profile is altered in a variety of medical conditions (e.g., cardiovascular and neurodegenerative) [52]. This justifies the strict inclusion criteria applied in the present review: studies including only healthy subjects or diagnosed MDD. Additionally, external lifestyle-related factors should be carefully monitored, as they may impact circulating EVs [122]. For instance, EEVs were previously shown to respond to high-fat meals [123], and PEVs were shown to respond to whole-body heat stress [124]. In fact, some studies in Table 3 specified a fasting period prior to blood collection [111,113] or even the absence of PE for a certain period [113]. The taken antidepressants may be also a confounding factor. Additionally, PE training protocols (type, intensity and volume) can also differentially modulate depression and EV outcomes similarly to their impact in cardiopulmonary adaptations, which were previously discussed in detail by others [51,52,74]. Another important issue is the demographic characteristics of the enrolled subjects. For example, although sex-specific responses were also reported for EV basal levels [55,95], these studies failed to describe age-specific responses. Finally, the problems of EV isolation, purification, identification and characterization methodologies and their impacts on measure outcomes are also thoroughly reviewed elsewhere [51,52,62,74]. The major guidelines in the field strongly recommend that a combination of quantification techniques is required to robustly characterize circulating EVs. In line with this, one of the trials in Table 2 reported different achievements regarding circulating EV quantification using two different techniques (NTA and FC) [99]. Overall, this section aimed to highlight the presence of confounding factors that must be carefully considered both in the search for potential depression diagnostic biomarkers and in the attempt to unravel putative mechanisms underlying PE-induced adaptations.

## 5. Future Directions: PE–EVs–Depression

**Figure 3:**
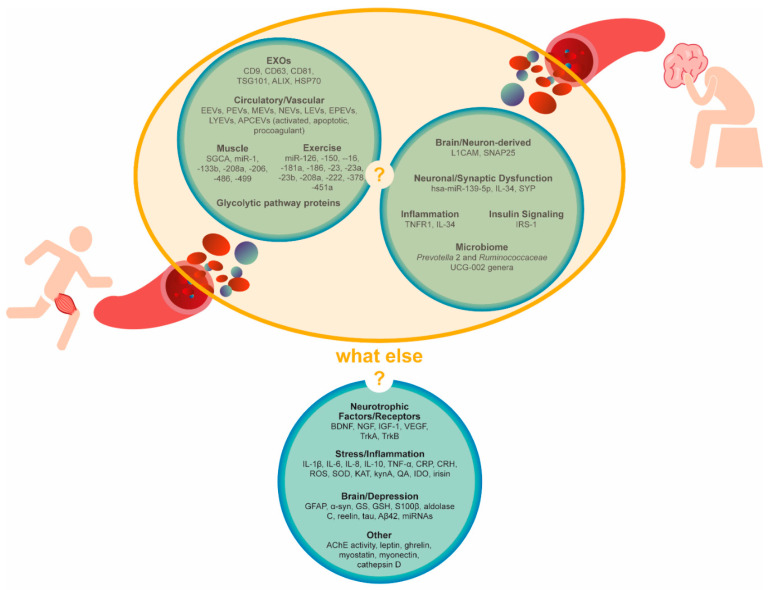
Circulating extracellular vesicle signatures after aerobic physical exercise or in the context of depression. Are they connected? Other candidate biomarkers. Circulating candidate biomarkers were suggested by the literature on physical exercise, inflammation or depression as either EV-cargoes or cell-free. AChE, acetylcholinesterase, APCEVs, antigen-presenting cell-derived vesicles, Aβ42, amyloid β42 BDNF, brain-derived neurotrophic factor, CRH, corticotropin-releasing hormone, CRP, C reactive protein, EEVs, endothelial-derived vesicles, EPEVs, endothelial progenitor cell-derived vesicles, EVs, extracellular vesicles, EXOs, exosomes, GFAP, glial fibrillary acidic protein, GS, glutamine synthetase, GSH, glutathione, HSP70, heat-shock protein 70, IDO, indoleamine 2,3-dioxygenase, IGF-1, insulin-like growth factor 1, IL, interleukin, IRS-1, insulin receptor substrate, KAT, kynurenine aminotransferase, kynA, kynurenic acid, LEVs, leucocyte-derived vesicles, LYEVs, lymphocyte-derived vesicles, L1CAM, L1 cell adhesion molecules, MEVs, monocyte-derived vesicles, NEVs, neutrophil-derived vesicles, NGF, nerve growth factor, PEVs, platelet-derived vesicles, QA, quinolinic acid, ROS, reactive oxygen species, S100β, S100 calcium-binding protein β, SNAP25, synaptosomal nerve-associated protein 25, SOD, superoxide dismutase, SYP, synaptophysin, TNF-α, tumor necrosis factor α, TrkA, tropomyosin receptor kinase A, TrkB, tropomyosin receptor kinase B, TSG101, tumor susceptibility 101, VEGF, vascular endothelial growth factor and α-syn, α-synuclein.

Although the meta-analysis and guidelines strongly recognize PE as an effective therapeutic strategy for depressive disorders, the prescription of PE remains elusive. We believe that the lack of knowledge regarding its underlying neurobiological mechanisms may justify this scenario. This reinforces the need for more detailed investigation on the possible mediators. In fact, the present review aims to encourage and provide some guidance for scientific research towards that direction. Although the EV research field is relatively novel, some strong data is already emerging. Overall, Table 1 shows that regular PE improved depressive symptoms in the depressive population. Table 2 evidences the modulation of circulating EVs by PE in healthy subjects. Although the link between depressive disorders and altered circulating EV signature is the least supported, it is starting to be unraveled (Table 3). In light of the current evidence, we still do not know if PE-triggered EVs contribute to the antidepressant effect of PE. However, the combination of the studies presented in Table 2 and Table 3 support our hypothesis (yellow circle illustrated in Figure 3), whereby circulating EVs contribute to the biological adaptations to PE in depression. Indeed, PE seems to change the concentration of circulating EVs and their cargo signatures (mostly protein and miRNA markers), possible mediators of vascular, metabolic, muscle, immune, inflammatory and neuronal adaptations during PE. Inflammation, particularly, seems to be a common mechanism in the PE-induced EV mechanism of action and in PE-induced positive effects in depression. Importantly, candidate biomarkers for depressive disorders often include inflammatory markers that are potentially carried by circulating EVs.

Finally, we encourage future studies designed to unveil the specific role of circulating EVs in tissue crosstalk, namely muscle-to-brain, and periphery-to-brain and brain-to-periphery signaling. Furthermore, we add additional candidate biomarkers to the ones summarized in Table 2 and Table 3 and illustrated in Figure 3 that were either discussed throughout the manuscript or extensively described elsewhere [44,45,46,64,65,66,68,72,75] that we consider as putative mediators of depression adaptations to PE (what else?, Figure 3). Those factors range from PE-induced exerkines (e.g., myostatin and, myonectin) to brain-derived protein markers (e.g., GFAP, α-synuclein (α-syn) and glutamine synthetase (GS)) and, also, include neurotrophic factors/receptors (e.g., BDNF, nerve growth factor (NGF), insulin-like growth factor 1 (IGF-1), vascular endothelial growth factor (VEGF), tropomyosin receptor kinase A (TrkA) and tropomyosin receptor kinase B (TrkB)); signaling mediators of stress and inflammation (e.g., interleukins/cytokines, C reactive protein (CRP), corticotropin-releasing hormone (CRH), reactive oxygen species (ROS), superoxide dismutase (SOD), kynA, indoleamine 2,3-dioxygenase (IDO) and irisin); depression-associated miRNAs and proteins (e.g., aldolase C, reelin, tau and Aβ42); lysosomal proteases (e.g., cathepsin D); hormones involved in energy balance (e.g., leptin and ghrelin) and cholinergic mediators (e.g., acetylcholinesterase (AChE)). The assessment of those markers within isolated circulating EVs, in the context of both regular PE practice and depression, may provide a more comprehensive picture of the underlying signaling mechanisms. Ultimately, specific EV signatures could be translated into future diagnostic and/or therapeutic biomarkers. Finally, the uniqueness of EV miRNA and protein responses in certain physiologic (e.g., PE) or pathologic conditions (e.g., depression) strongly suggests that EVs must be further explored for a better understanding of their role in the body’s homeostasis.

## 6. Conclusions

Aerobic physical exercise is an evidence-based and attractive therapy for depressive disorders. This non-pharmacological strategy can modulate inflammation, which is a critical factor in depressive disorder pathophysiology. Therefore, herein, we argue that the effect of PE in depression management may be mediated through altered circulating EV signatures, which are associated with decreased systemic inflammation. The exciting, yet scarce, body of evidence on this complex network warrants more research, particularly in the context of regular aerobic PE practice in depressive populations. While EVs have been implicated in cardiovascular and neurodegenerative diseases, research regarding their role in depression remains also limited. This review provides new insights and guidance on candidate EV cargo biomarkers for future studies aiming to extend the knowledge on depression adaptations to PE.
